# Theory-based metrological traceability in education: A reading measurement network

**DOI:** 10.1016/j.measurement.2016.06.036

**Published:** 2016-10

**Authors:** William P. Fisher, A. Jackson Stenner

**Affiliations:** a LivingCapitalMetrics.com, Sausalito, CA, USA; b BEAR Center, Graduate School of Education, University of California, Berkeley, CA, USA; c MetaMetrics, Inc., Durham, NC, USA; d School of Education, University of North Carolina, Chapel Hill, NC, USA

**Keywords:** Psychometrics, Reading ability, Education, Predictive theory, Unit standards, Test equating, Social studies of science, Commercial applications, Traceability

## Abstract

Huge resources are invested in metrology and standards in the natural sciences, engineering, and across a wide range of commercial technologies. Significant positive returns of human, social, environmental, and economic value on these investments have been sustained for decades. Proven methods for calibrating test and survey instruments in linear units are readily available, as are data- and theory-based methods for equating those instruments to a shared unit. Using these methods, metrological traceability is obtained in a variety of commercially available elementary and secondary English and Spanish language reading education programs in the U.S., Canada, Mexico, and Australia. Given established historical patterns, widespread routine reproduction of predicted text-based and instructional effects expressed in a common language and shared frame of reference may lead to significant developments in theory and practice. Opportunities for systematic implementations of teacher-driven lean thinking and continuous quality improvement methods may be of particular interest and value.

## 1 Introduction

Metrology connects measurement applications across industrial, scientific, and practical tasks separated by space and time. Significant fractions of many nations’ economic productivity are invested in ensuring traceability to standards for various units of measurement. The human, social, environmental, and economic value of the returns on these investments depends on the transparency of the measures and their integration into a wide range of decision processes at multiple organizational levels. Huge resources are required to create and maintain technologically produced effects, such as volts, seconds, or meters, with the primary return on those resources being the illusion that the effects seem to be products of nothing but completely natural processes occurring with no human intervention.

New insights into how cognitive, social and technological resources aid in creating shared cultural frames of reference have emerged from close critical study of historical and contemporary scientific modelling and metrological practices. From this perspective, science is not qualitatively different from everyday ways of thinking and relating, except in more deliberately extending laboratory processes into the world as distributed cognitive systems supporting a range of associated problem-solving methods [1], [2], [3], [4], [5]. Of particular interest here is the linking of specific ways in which organizations align and coordinate their processes and relationships relative to technical developments and expectations. A positive result of adopting this point of view is recognition of the value of previously obscured accomplishments in, and opportunities for, advancing the quality of research and practice in psychology and the social sciences. An illustrative example is found in the scientific modelling and metrological practices informing integrated reading assessment and instruction in education.

### 1.1 Transparent instruments, invisible production

By definition, metrologists are doing their jobs best when no one knows they are there. Experimental scientists, for instance, may take little notice of their instrumentation until it breaks down or does not conform to expected standards. The general public and researchers in psychology and the social sciences are, then, also largely unaware of the resource-intensive work involved in establishing uniform unit standards and traceability to them [3], [4], [6], [7].

The uniformity of the various phenomena described by natural laws allows scientists the convenient efficiency of not needing to specify scale units in statements of laws. Force equals mass times acceleration in kilograms, Newtons, and meters just as well as in pounds, poundals, and feet. The ability to skip over uniform details supports a division of labour in science that separates theoretical work from the calibration of instruments and both of these from the use of theory and instruments in experiments [8].

The convenience of separating theoretical, experimental and instrumental concerns has its drawbacks, too. Not knowing when or how reference standard units are established reinforces unexamined metaphysical assumptions—such as the idea that the universe or nature is inherently and innately numerical, quantitative, or mathematical—that rarely become explicit objects of attention.

The effect of these presuppositions is significant. Huge social, industrial, and economic efficiencies are gained by universal consensus on the facts of complex phenomena like electricity, temperature, distance, mass, and time. Though the dynamics of that consensus are complex and sometimes counterintuitive [8], making quantities seem natural is a cultural achievement of the highest order.

The advancement of science is put at risk when the historic and historical mathematical understanding of scientific objects is reified as unquestioned and unquestionable. Two questions emerge here: (1) how did the natural sciences succeed in making quantities seem so thoroughly natural [3], [4], [9], [10], [11], [12], and (2) how might the social sciences learn from those successes? Recent advances in reading measurement embody important lessons in this regard for the social sciences.

### 1.2 Shortsightedly focusing attention on the local measurement outcome

The technical processes of measurement were historically cut out of the picture of science by the positivist focus on empirical observation, as well as by the later anti-positivist focus on theoretical constraints on observation [8]. Sometimes this omission was literal and deliberate, as when a woodcut of a laboratory scene printed in its entirety in one place is trimmed in a later publication to exclude the means by which a technical effect was produced [12]. Other times the omission was metaphorical, as when technical processes were illustrated in summary form by angelic cherubs producing effects by means of divine intervention [12].

Transparency in measurement is a two-edged sword. Wide access to comparable measures is achieved only to the extent that technical complexities can be ignored. This point was emphasized by Whitehead [13], who observed that “Civilization advances by extending the number of important operations which we can perform without thinking about them” (p. 61). But what happens when those making these advances do not record—or do not themselves fully understand—*how* they extended the number of important operations that can be performed by persons unversed in their technicalities?

In his study of the geometric assumptions Galileo employed in his physics, Husserl [14] was sensitive to the ways in which a hidden agenda set priorities. Like Galileo, we find ourselves in a situation, in accord with the philosophical problems attending measurement, in general, where

Metrology has not often been granted much historical significance. ... Intellectualist condescension distracts our attention from these everyday practices, from their technical staff, and from the work which makes results count outside laboratory walls [6].

Researchers in the natural sciences make use of commercially available precision tools calibrated to universally uniform reference standards, standards capitalizing on the value of invariant laws. Transparent measures communicated in a network sharing common values situates metrology’s often unrecognized historical significance in a complex overall context offering important lessons for psychology and the social sciences [1], [2], [3], [4], [5], [6], [7], [8], [9], [10], [11], [12]. The culture of science rewards a mix of convergent, divergent, and reflective thinking in ways that have proven their productivity and inform a vital culture of ongoing innovation [8], [9], [11], [12], [15].

### 1.3 Consequences for psychology and the social sciences

But in the social sciences, the lack of metrological institutions, methods, and traditions, and the associated absence of the intercalated disunity of distinct theoretical, experimental, and instrumental communities observed by Galison in the natural sciences [8], has been catastrophic. As social scientists have long recognized for themselves [16], [17], [18], mainstream research methods and statistical models employ scale-dependent ordinal data in a search for a kind of significance that is often irrelevant to and even antithetical to the production of new knowledge. Even when regularities akin to natural laws are sought and found in psychological and social phenomena [19], [20], [21], [22], [23], results are typically assessed in the language and methods of statistics rather than of measurement and metrology, meaning the focus is on data analysis and not on theory development or the calibration of instruments traceable to a standard unit. The human, social, economic, and scientific consequences of this failure to coordinate and balance convergent, divergent, and reflective field-defining activities are profound. Ideas on how such activities might be organized in education have recently been proposed [24].

The lack of institutions and traditions concerning metrological traceability and standards in psychology and the social sciences may have more to do with broad and deep cultural presuppositions than with an actual lack of a basis for them in evidence. After all, what systematic program of experimental evaluation has ever irrefutably established that uniform metrics based in lawful regularities are impossible in psychology and the social sciences? Evidence indicates that provisional possibilities exist in some circumstances [19], [20], [21], [22], [23], [24].

## 2 Metrological traceability for reading measurement

The longstanding need to provide students with reading challenges appropriate to their reading abilities is usually approached in terms of general curricular structures, and teacher training and experience. Theory has not been of significant interest [25], [26]. Rasch’s development of a new class of measurement models in the 1950s was an important step forward in improving the quantification of reading ability [25]. This research led to improvements in the matching of readers to text.

When Rasch’s concept of specific objectivity (the modelled independence of the ability and difficulty parameters, as shown in Eq. (1)) as it was obtained in local measures was combined with a general predictive theory of English text complexity in the 1980s, following the work of Stenner and colleagues [27], [28], [29], the stage was set for the efficient creation of a network of reading measurement instruments calibrated in a common unit. By the late 1990s, all of the major high stakes English reading tests in the U.S. had been brought into the system. These are today complemented by the hundreds of thousands of books, tens of millions of short articles and hundreds of millions of readers that have been brought into the system in the intervening years.

In this system, reader abilities and text complexities are measured in the same unit. The scale ranges from below 200 for beginning readers to over 1600 for very high level readers and texts. Knowing the text measure of a book and the reader’s measure predicts the degree to which the book will be comprehensible to the student.

**Fig. 1:**
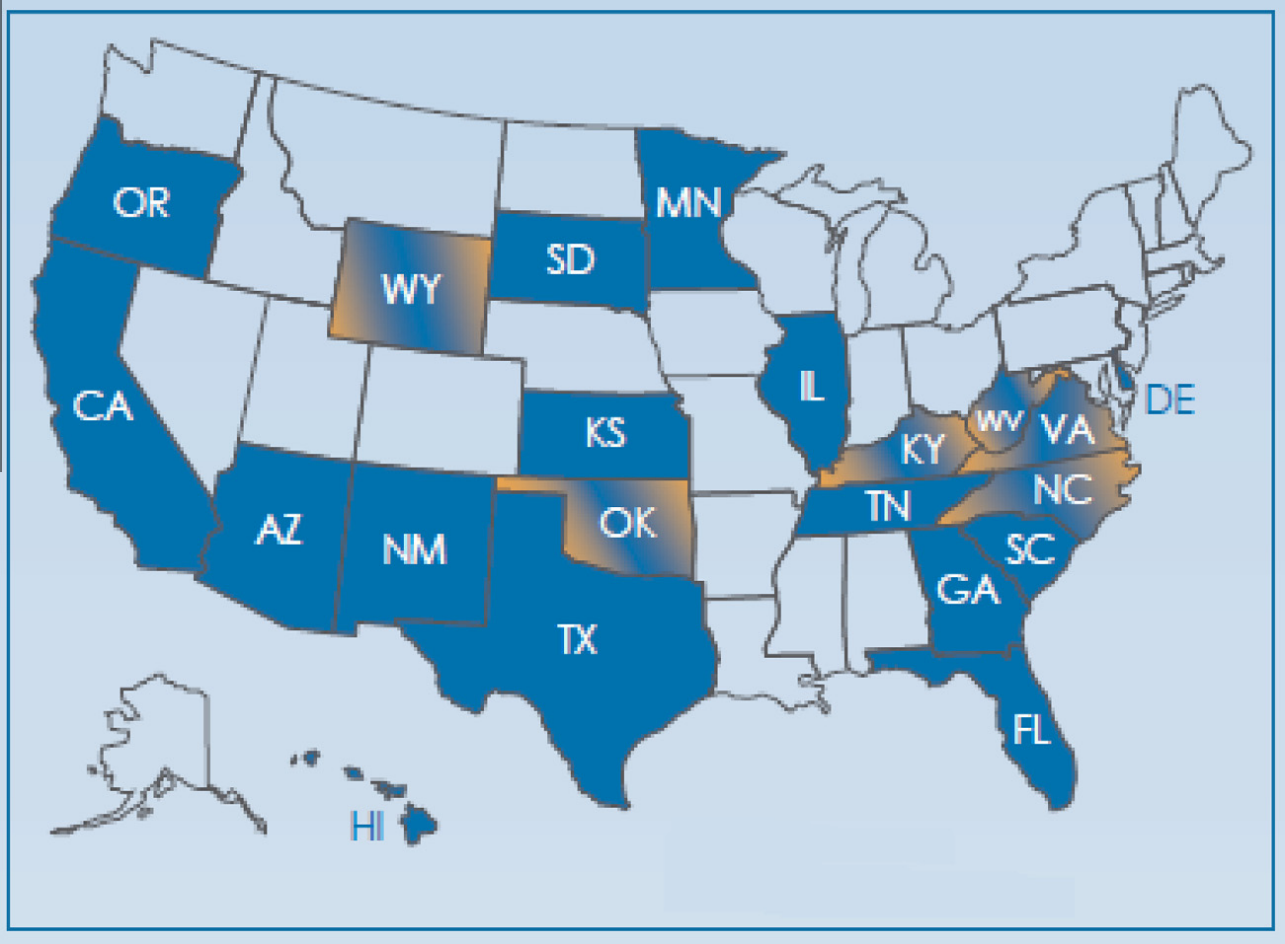
Map of U.S. states employing a common unit of reading measurement (two tones indicate common units in use for both reading and mathematics).

More than 30 million measures annually are reported in the U.S. from state and commercial assessments, and from classroom reading program assessments, in a common unit of measurement [29]. The 21 U.S. state departments of education that have formally adopted this unit for use are shown in the map in Fig. 1.

Traceability to the common unit is determined via both empirical, data-based equating studies and theory-based text analyses [29]. Additional features of the system include electronic tools integrating instruction and assessment for mass customized diagnostics [30], and others charting growth in reading ability relative to college and career readiness [31], [32]. Establishing this network of comparable assessments required formal relationships with book and test publishers, teachers, schools and school districts, state departments of education, and psychometric researchers. Furthermore, a new array of material practices was needed to give all the parties involved ongoing and verifiable confidence in the theory. Though great efficiencies stood to be gained, credibility demanded a cautious approach to their implementation. Formal documentation of the birth of this traceability system would be a valuable contribution to the sociotechnical qualities of education.

## 3 Implications for psychology and the social sciences

In 1965, the National Academy of Sciences published a report articulating common assumptions as to the sequence of events supposed to take place in the development of new instrumentation [33]. Four stages were identified:

(1) discovery of suitable means of observing some phenomenon,
(2) exploration of this phenomenon with special, homemade instruments or commercial prototypes,
(3) widespread use of commercial instruments,
(4) routine applications of the instrument to control industrial production as well as research.

Textbook assumptions and presentations of this sequence have indoctrinated researchers in the human sciences to believe, mistakenly, that this is the normal sequence of events. Because hardly anyone is involved in every part of the process, unexamined assumptions cohere into a “just-so” narrative that says more about cultural expectations than about historical complexities. Scientists and non-scientists alike accept this story, against the grain of actual events. Rabkin [33] points out that

this scheme seems to be at variance with much of the evidence in the history of science. It has been shown that the integration of instruments has been rarely due to the demand on the part of the researcher. Rather it occurs through vigorous supply of advanced instruments on the part of the industry. The company that proposes these four stages in the report has itself had experience when stages 3 and 4 occur in the reverse order and, moreover, stage 4 is by far the most decisive factor in the development of new instrumentation.

The “vigorous supply of advanced instruments”, and not demand, also characterizes the introduction of popular electronic appliances. Just as Rabkin points out has been the case in research, there was little or no clamour among the public for telephones, televisions, faxes, the Internet, microwaves, blenders, or cell phones before they were developed and introduced.

Scientists and the public both tend to think of instrumentation only as tools employed in the service of the individuals who use them. This perspective is at odds with the historical evidence as well as with philosophers’ observations, such as, for instance, Thoreau’s realization that humanity has become the tool of its tools [34] and Nietzsche’s insight that the victory of science is better cast as a victory of method over science [35].

This alternative perspective is important because, in the history of science, theory follows from extensive experience with instruments more often than instruments are designed and built from theoretical projections. Standardized and commercially available instrumentation make possible the predictable and routine reproduction of scientific effects essential to the conduct of controlled experiments—and so also to the development of precise and accurate theoretical predictions. As stated by Price,

Historically, we have almost no examples of an increase in understanding being applied to make new advances in technical competence, but we have many cases of advances in technology being puzzled out by theoreticians and resulting in the advancement of knowledge. It is not just a clever historical aphorism, but a general truth, that ’thermodynamics owes much more to the steam engine than ever the steam engine owed to thermodynamics.’ ...historically the arrow of causality is largely from the technology to the science [36].

In the context of reading measurement, the repeated reproduction of consistent results following the work of Rasch and others led to the Anchor Test Study in the 1970s [37]. This study equated seven major reading tests in the U.S. and involved over 350,000 students in all 50 states. But the purely empirical basis of the calibration and the lack of predictive theory meant that the value of the common unit of measurement was lost as soon as new items were added to the tests, which was immediately.

A plain feature of the equated test results, however, was the similarity of the items from different tests that calibrated in the same locations. The stability of this phenomenon may not surprise anyone able to read, but its practical application in a predictive theory relating text complexity, comprehension rates, and reading ability was difficult to achieve [38].

## 4 Theory for reading measurement

The ability to read is fundamental to education, and it is accordingly tested and measured more often than any other subject area. The index to the eighteenth edition of the Buros Mental Measurements Yearbook [39] includes over 140 tests with the word “reading” in their titles. This count does not include tests focused on vocabulary or word meaning, which are also numerous.

Though the issues are complex, literacy remains essential to productivity in the global economy [40]. The need for effective and efficient reading education will only intensify as communication, teamwork, and information management are increasingly demanded as basic skills [41].

And despite the longstanding fundamental importance of reading as the tool most essential to learning, reading research remained atheoretical until 1953, and interest in a unified theory of reading is a relatively new phenomenon [25], [26]. Further, in the years since 1953, available reading theories have not generally been used to inform the design or interpretation of assessments of reading ability [25].

Though it may seem counterintuitive, this failure to apply theory in the course of empirical measurement research is not unusual, nor is it restricted to reading research. On the contrary, measurement technologies in the natural sciences have historically been developed through socially-contextualized trial-and-error solutions to practical engineering problems, such as consistent, stable results, and not directly from theoretical principles [3], [4], [5], [6], [7], [8], [33], [36], [42]. Theory generally comes later, after researchers have had the opportunity to employ standardized technologies in the routine and repeated reproduction of a controlled phenomenon. Only then do applicable general principles emerge as useful insights that can be fed back into technical refinements.

### 4.1 Syntactic and semantic elements

In the same way putting things in words reduces an infinite variety of ways an experience might be expressed into a particular set of words expressed in a particular language, science reduces the infinite variations that phenomena exhibit to simpler models. The truth of the models is less an issue than their usefulness [43], [44]. Simplification is usually achieved only in contexts that respect constraints and accept limited goals. The efficiency and power obtained when useful tools can be created, however, confers great value on a simplified process.

In the 1950s, Rasch’s parameter separability theorem, concept of specific objectivity, and models useful in practical measurement applications combined in an important step forward in educational measurement [45]. These developments were followed by Wright’s introduction of improved estimation algorithms, model fit tests, and software in the 1960s, along with his vigorous championing of Rasch’s ideas [46]. By the 1970s, enough data from reading tests had been successfully fit to Rasch models in the U.S. to support the viability of the Anchor Test Study [37]. Success in this large project and additional research predicting item difficulties on the Peabody Vocabulary Test and the Knox Cube Test (a measure of short term memory and attention span) [27], [28], led to a new effort focused on developing explanatory theory for reading.

Reading theories build on the fact that all symbol systems share two features: a semantic component and a syntactic component. In language, the semantic units are words. Words are organized according to rules of syntax into sentences [47]. Semantic units vary in familiarity and the syntactic structures vary in complexity. The readability of a text passage is dominated by the familiarity of the semantic units and by the complexity of the syntactic structures used in constructing the message. Many readability equations therefore use a two-variable equation to forecast text difficulty. The word-frequency and sentence-length measures combine to produce a regression equation, known as a construct specification equation [27], [28]. This equation provides a theoretical model evaluated in terms of the proportion of the variance of reading comprehension task difficulties (or, more recently, the means of specification-equivalent ensembles of item difficulties, following Gibbs [48]) that can be explained as plausibly structured by causal relationships [49].

### 4.2 The specification equation

One approach to such a specification equation first employs the mean of the logarithm of the frequencies with which words in a text appear in a 550-million word corpus of K-16 texts. More specifically, the log frequency of the word family, which is more highly correlated with word difficulty, comprises one term in the equation. Word families include the stimulus word, all plurals, adverbial forms, comparatives, superlatives, verb forms, past participles, and adjectival forms. The frequencies of all words in the family are summed and the log of that sum is used in the specification equation.

The second term of the specification equation is the logarithm of the text’s mean sentence length. This parameter is operationalized simply by counting and averaging the number of words in each sentence.

The theoretical logit is then a function of sentence length and word frequencies in the language stated in the specification equation:

(1) $$\text{Reading difficulty}(\text{or readability})=\text{A}*\log(\text{MSL})-\text{B}*\overset{‾}{\log(\text{WF})}+\text{C}$$

where MSL is the mean sentence length and WF is the word frequencies. Log(MSL) and the mean log(WF) are used as proxies for syntactic complexity and semantic demand, and the coefficients are drawn from the empirical regression study [50]. Research is continuing into the decimal place significance of the coefficients and measurement uncertainty for the values of A (9.82247), B (2.14634), and C (a constant). The resulting logits are then scaled as follows:

(2) (logit+3.3)∗180+200

The relationship of word frequency and sentence length to text readability was investigated in research that extended a previous study on semantic units [28]. The original study found success on items at about −3.3 logits as indicating the earliest reading ability, and set that level at 200. A practical top to the scale for the end of high school was at 2.3 logits, and this was set 1000 units higher, to 1200. There is no upper limit to the scale, but text measures above 1600L are rare.

In this unit, when student and text measures match, a 75% comprehension level is expected. A student with a measure of 500L is expected to answer correctly 75% of the questions on an assessment made from any text that also measures 500L, within the range of uncertainty. The 75% comprehension rate differs from the default rate of 50% comprehension usually associated with matching measures and calibrations. Though the lowest uncertainty is associated with the 50% rate, teachers find that instruction has a firmer basis in student confidence when success is more likely. For this reason, the relation of ability to difficulty was shifted from 50% to 75% comprehension.

The uncertainty (standard error) of the individual measures [51] is

(3) $$\text{SE}=\text{X}*{\left[\text{L}/(\text{r}(\text{L}-\text{r}))\right]}^{\wedge }(1/2)$$

which is the square root of the test length L divided by the count correct r times the L − r count incorrect, times an expansion factor X that depends on test width. This logit is then converted to the standard unit. A standard unit uncertainty for a well targeted 36-item test measuring with an uncertainty of about .40 logits is the original logit range of 2.3 − (−3.3) = 5.6 divided into the 1000L range, times .40, which comes to about 71L.

The analysis reported in the original study [28] involved calculation of the mean word frequency and the log of the mean sentence length for each of the 66 reading comprehension passages on the Peabody Individual Achievement Test. The observed difficulty of each passage was the mean difficulty of the items associated with the passage (provided by the publisher) converted to the logit scale.

A regression analysis based on the word-frequency and sentence-length measures produced a regression equation that explained much of the variance found in the set of reading comprehension tasks. The resulting correlation between the observed logit difficulties and the theoretical calibrations was 0.97 after correction for range restriction and measurement error [50].

The regression equation was further refined based on its use in predicting the observed difficulty of the reading comprehension passages on eight other standardized tests (see Table 1). Repeated and ongoing comparisons of theoretically expected calibrations with data-based estimates produced from test data analysis provide continually updated validity evidence.

The regression equation links the syntactic and semantic features of text to the empirically determined difficulty of text. That link, in turn, is reproduced across thousands of test items and millions of examinees.

In applications the consistent display of the link over time provides a basis for using the equation to perform theory-based calibrations of test items and texts, thus rendering empirical calibrations necessary only as checks on the system.

This specification equation joins together previously separated but analogous developments in measures of information. Hartley’s [52] log(N) measure of information content (the number of signs in a message), for instance, is akin to the sentence length parameter in equation (1). Similarly, the word frequency parameter is akin to Shannon’s [53] classic expression p ∗ log(p), where more information is implied by a word’s greater rarity in the language. Including Shannon’s extra p (multiplying the log of the probability of observing a sign by that probability) indicates the entropy of the area under the curve in the logistic ogive [54].

## 5 Benefits of metrological comparability

A wide range of applications for text measures have emerged in recent years [31], [32], [55], [56]. Measures of information content are taking a wide range of forms, many involving entropy. These statistical approaches tend to be dependent on particular data sets and algorithms. Little, if any, attention is put into identifying and implementing an invariant unit of measurement, or into designing and maintaining a metrological network of instruments traceable to such a unit.

The benefits of metrological comparability for measuring reading ability extend from the advancement of education science’s basis in theory to practical quality improvement methods in schools and classrooms [57]. The natural sciences and the monetary economy both enjoy a degree of efficiency in their markets for the exchange of information and prices. This efficiency stems in large part from the existence of rules, roles, and responsibilities [1], [2], [3], [4], [5], [6], [7], [8], [9], [10], [58] associated with the institutionalization of common units of measurement, such as meters, grams, degrees Celsius, or dollars.

Suppliers, manufacturers, marketers, accountants, advocates, and customers are able to better coordinate and align their investments in physical capital when information systems employ common languages. Similar kinds of coordinations can be expected to emerge as teachers, researchers, and psychometricians establish firmer expectations for educational outcomes and the exceptions that prove (in the sense of test) the rules. For instance, quality circles will facilitate the exchange of instructional outcome information across classrooms, grades, and schools in ways not possible with test scores reported in traditional percentages correct. Curriculum publishers are already developing individualized reading instruction modules that integrate assessment information in ways that make student learning trajectories portable across proprietary tests, schools, and countries.

## 6 Discussion

Projected comprehension rates should not be the only factor influencing text selection. To make the quantified measure the sole determinant of a curricular decision would be analogous to reducing a table to its physical dimensions when its colour, style, or sentimental or historical value might also be relevant.

Initial efforts at deploying the unit of measurement quickly encountered a chicken and egg question from book publishers: why should they adopt the unit as a means of indicating the text complexity of their books and articles if there were no schools or students prepared to take advantage of that information? Conversely, state departments of education and school districts asked, why should they be interested in a universally uniform measure of reading ability if there were no books or articles to match with students’ ability measures?

The solution arose when one publisher incorporated the unit in their own system, involving both a reading curriculum and a reading assessment system. This coordinated reader-text matching made the link to the unit more attractive to testing agencies, who could now point to an additional use for their results; to book publishers, who now were assured of a population of students with measures to match with their books; and to state departments of education and school districts, who could now effectively put the matching system to work.

The English-based system is in use in the U.S., Canada and Australia (with applications emerging in New Zealand, South Africa, and England), and in ESL applications in Korea, Japan, Malaysia, Hong Kong, and elsewhere in Asia. A Spanish system for matching readers and texts in the same unit is in use in Mexico and the Philippines. Researchers in various parts of the world are exploring possibilities for expanding the reader-text matching system to Mandarin, French, Arabic, and other languages.

**Fig. 2:**
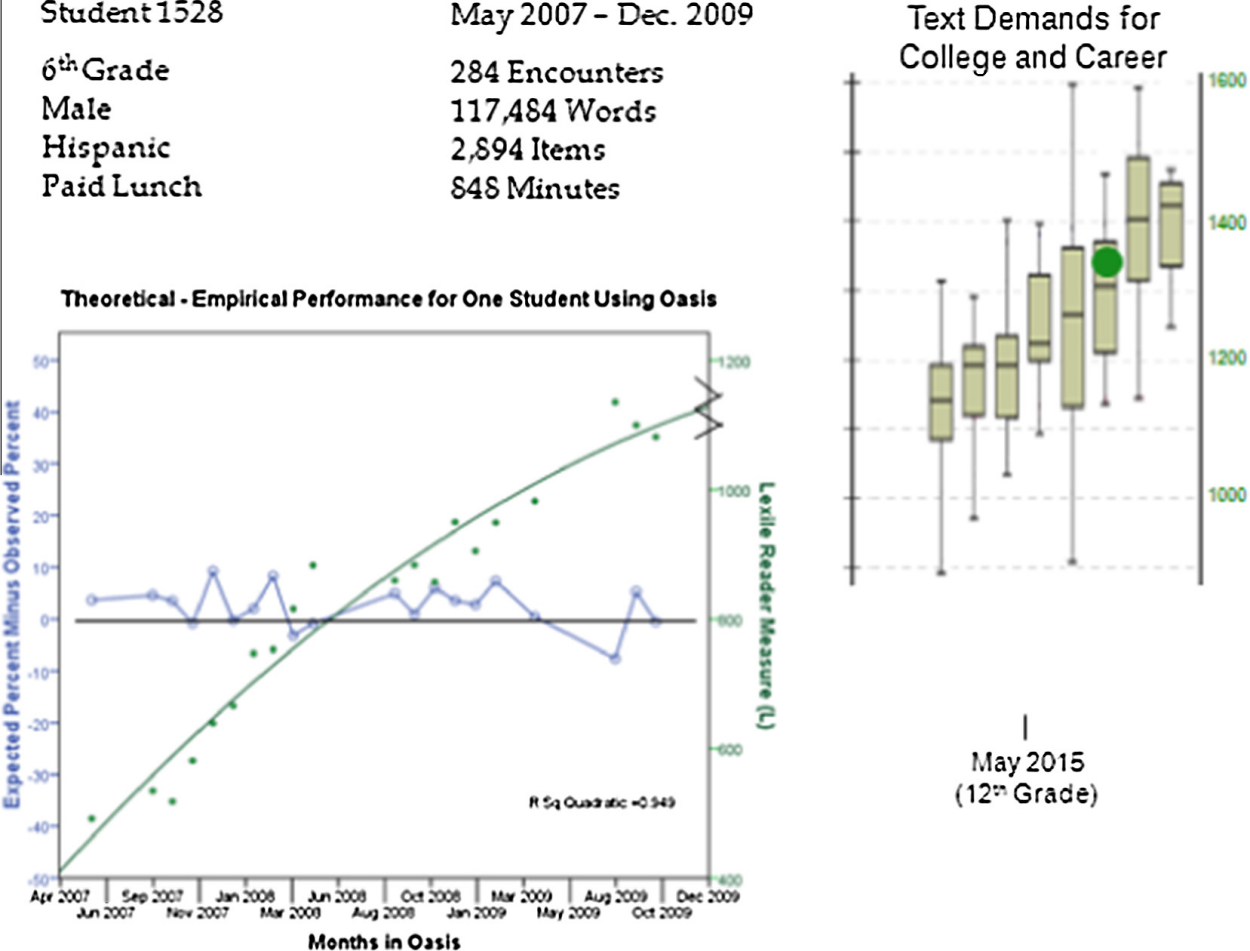
Individual student online reading measurement tracking system report, text domains from left to right are: High School (11–12), SAT I ACT AP, Military, Citizenship, Workplace, Community College, University, Graduate Record Exam.

Educational textbook and curriculum publishers have developed online software applications for tracking individual student growth in reading ability. A report from one such system is shown in Fig. 2. The value of repeated measures of a student over time and across texts is evident in the growth trajectory and the expected convergence of the student’s ability with the reading demands of adult life.

**Fig. 3:**
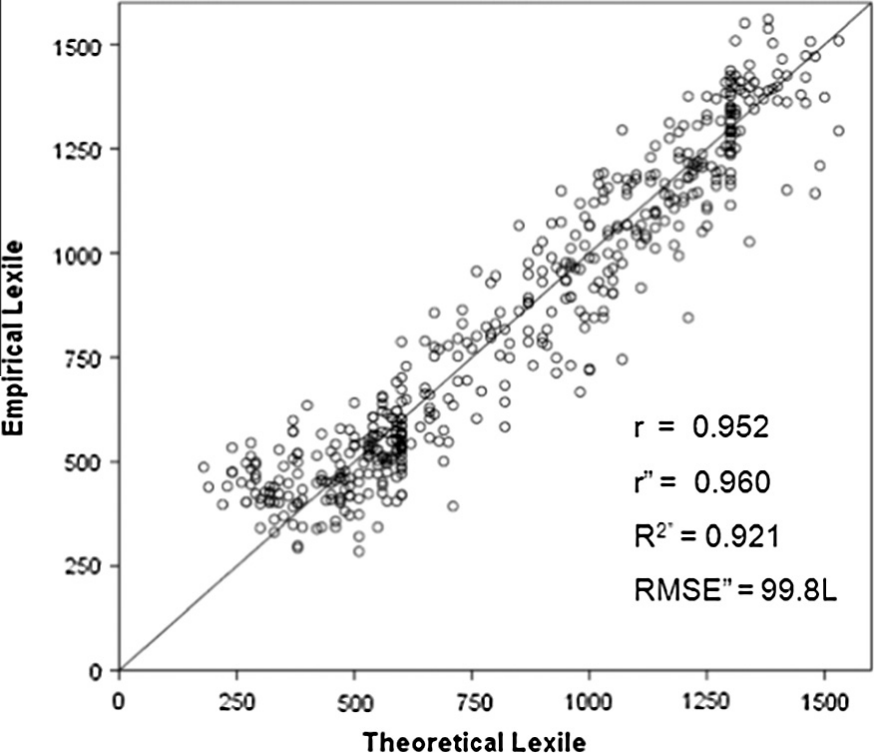
Empirical vs. theoretical Lexile text complexity estimates.

Fig. 3 shows the relationship between expected and observed text complexity measures in the online system. This plot illustrates the power of theory. Traceability to the standard unit is achieved not only by estimating student reading ability measures from data, but by gauging text complexity from its syntactic and semantic makeup. Given theory-based estimates of item difficulty, items can be adaptively selected for custom-tailored individualized administration, and those students’ measures may then be estimated from their comprehension rates relative to the scale values of those items.

The specification equation operationalizes Rasch’s notion of a frame of reference in a way that extends the frame beyond the specific objectivity obtained in the context of a particular test or set of equated tests to an indefinitely large collection of actual or virtual instruments, students, and texts. Theory-based instrument calibration eliminates the need to use data to both calibrate instruments and measure persons. The pay-off from using theory instead of data to calibrate instruments is large and immediate. When data fit a Rasch model, *differences* among person measures are, within the limits of uncertainty and response consistency, free of dependencies on other facets of the measurement context (i.e., the differences are specifically objective). When data fit a causal or theory-enhanced Rasch model, *absolute* person measures are free (again, within the limits of uncertainty and response consistency) of the conditions of measurement (items, occasions, etc.) making them objective beyond the limits of a specific frame of reference tied to local samples of examinees and test items [49], [50]. In the theory-referenced context, person measures are individually-centred statistics; no reference to another person(s) figures in their estimation.

One of the most important uses of reading test scores is to predict how a reader will perform on non-test tasks. For example, imagine that first year college textbooks are virtual reading tests with item calibrations provided by the specification equation. Arbitrarily, but usefully, fixing a success rate on the virtual items for each textbook enables solving for the reader measure needed to correctly answer that percentage of those items. The individual reader’s measure is then interpreted relative to the text complexity measure for each text in the freshman book bag. If the likely success rate in correctly answering the virtual items is high, so is the expectation of having the reading skills needed to complete the first year of college.

High school graduates’ reading measures can thus be compared to college text demands and a reasoned prediction can be made as to the likelihood of having the reading level needed for first year completion. The efficiencies this system realizes from its use of validated predictive theory shows special promise as a tool for tracking reading readiness for post-secondary experiences in college, the work place, and the responsibilities of citizenship [31], [32].

## 7 Conclusion

Historians of science have repeatedly documented the roles in theory development played by researchers with hands-on experience with instrumentation, as when Kuhn [59] notes that seven of the nine pioneers in quantifying energy conversion processes were either trained as engineers or were working with engines when they made their contributions. Indeed, this attitude that an instrument can make a science was taken from physics into economics by both Stanley Jevons and Irving Fisher in their uses of the balance scale as a model of market equilibrium [60], [61].

But history shows that instruments alone are insufficient to the task of making a science. Furthermore, interestingly, equilibrium models have failed as guides to economic phenomena in large part because of problems in stochastic aggregation and variation in individual consumer behaviours [62]. In specific circumstances [1], [2], [3], [4], [5], [6], [7], [8], [9], [10], [11], [12], however, instruments providing consistent information expressed in a common language throughout interconnected nodes of a network, as with the reading measurement system described here, may serve as a medium for coordinating spontaneous individual behaviours and decisions over time and space.

The historical success of science increasingly appears to stem from its embodiment of evolving ecologies of this kind of data-theory-instrument assemblage. Current conceptualizations and institutional systems prioritizing centralized design, data analysis, and policy formation stand in paradigmatic opposition to this ecologizing perspective [63], [64], [65], [66], [67]. How will cultures of decentralized innovation, complex self-organization, and authentic engagement with substantive, meaningful processes emerge in education and the social sciences? The organic integration of theory, data, and instruments in institutional contexts sensitive to ground-up self-organizing processes requires systematic conceptualizations of measurement as a distributed process, where scientific fields, markets, and societies operate as massively parallel stochastic computers [66], [67]. Recent comparisons of engineering and psychometric perspectives on the possibility of such systems in education suggest a viable basis for such conceptualizations [68], [69], [70], [71], [72], [73]. Metrological traceability systems of this kind [24] will integrate qualitative progressions in learning defined by predictive theories of causal relations [49], construct maps [74], and associated item hierarchies in educational assessments generally. Systematically introduced infrastructural supports could effectively exploit the proven value of formative assessment [75] in a hopeful development for broadly enhancing educational outcomes via research and local quality improvement efforts.

## Figures and Tables

**Table 1:** Correlations of theory-based calibrations produced by the specification equation and data-based item difficulties.

Test	# of Questions	# of Passages	r_(OT)_^a^	R_(OT)_^b^	*R*^∗^_(OT)_^c^
SRA	235	46	.95	.97	1.00
CAT-E	418	74	.91	.95	.97
Lexile	262	262	.93	.95	.97
PIAT	66	66	.93	.94	.97
CAT-C	253	43	.83	.93	.96
CTBS-U	246	50	.74	.92	.95
NAEP	189	70	.65	.92	.94
Battery	26	26	.88	.84	.87
Mastery	85	85	.74	.75	.77

Totals	1780	722			
Means			.84	.91	.93

a r_(OT)_ = raw correlation between observed difficulties (O) and theory-based calibrations (T).

b R_(OT)_ = correlation between observed difficulties (O) and theory-based calibrations (T) corrected for range restriction.

c R^∗^_(OT)_ = correlation between observed difficulties (O) and theory-based calibrations (T) corrected for range restriction and measurement error.
